# Exploiting large language models for diagnosing autism associated language disorders and identifying distinct features

**DOI:** 10.1038/s41746-025-02133-9

**Published:** 2025-12-16

**Authors:** Chuanbo Hu, Wenqi Li, Mindi Ruan, Xiangxu Yu, Shalaka Deshpande, Lynn K. Paul, Shuo Wang, Xin Li

**Affiliations:** 1 Department of Computer Science, University at Albany, Albany, NY USA; 2 Lane Department of Computer Science and Electrical Engineering, West Virginia University, Morgantown, WV USA; 3 Department of Radiology, Washington University in St. Louis, St. Louis, MO USA; 4 Oregon State University, Corvallis, OR USA; 5 Humanities and Social Sciences, California Institute of Technology, Pasadena, CA USA

**Keywords:** Neuroscience, Biomarkers, Mathematics and computing

## Abstract

Diagnosing language disorders associated with autism is a complex challenge, often hampered by the subjective nature and variability of traditional assessment methods. In this study, we explored large language models (LLMs) to overcome the speed and precision obstacles by enhancing sensitivity and profiling linguistic features for autism diagnosis. This research utilizes natural language understanding capabilities of LLMs to simplify and improve the diagnostic process, focusing on identifying autism-related language patterns. We showed that the proposed method demonstrated improvements over the baseline models, with over a 10% increase in both sensitivity and positive predictive value in a zero-shot learning configuration. Combining accuracy and applicability, the framework could serve as a valuable supplementary tool within the diagnostic process for ASD-related language patterns. We identified ten key features of autism-associated language disorders across scenarios. Features such as echolalia, pronoun reversal, and atypical language usage play a critical role in diagnosing ASD and informing tailored treatment plans.

## Introduction

Autism spectrum disorder (ASD) is a developmental condition marked by difficulties in social interaction, restricted interests, and repetitive behaviors.^1–3^. The spectrum of ASD symptoms is broad, with communication difficulties often standing out as the most significant and impacting aspects of the disorder^4–6^. These symptoms vary across age groups. In adults, communication difficulties are especially pronounced, significantly affecting social integration and personal development^7,8^. Identifying these communication issues is crucial for effective intervention. Language disorders in adults with ASD include a wide range of issues, from the absence of speech to subtle impairments such as echolalia (repetitive use of phrases or sounds), pronoun reversal, and pragmatic language impairments^9–11^. These disorders can hinder effective communication and pose significant challenges in social and occupational settings. Accurate identification of language anomalies is essential for timely interventions.

While the Autism Diagnostic Observation Schedule, Second Edition (ADOS-2)^12^ is a gold standard for ASD diagnosis across various age groups. The ADOS-2 involves structured, standardized observation sessions, typically lasting around one hour, during which an examiner engages the patient in a series of conversational and interactive tasks. These sessions are designed to assess key areas of communication, social interaction, and behavior. For adults, the examiner analyzes the patient’s responses, verbal and non-verbal communication patterns, and social cues to identify markers of ASD. The diagnostic process relies on the examiner’s ability to interpret subtle interaction dynamics and communication behaviors, making the method highly reliant on clinical expertise. As a result, the approach can be both subjective and resource-intensive, posing challenges for scalability and consistency in assessments.

To address these challenges, researchers have turned to machine learning (ML) as a complementary or alternative diagnostic tool. ML models leverage multimodal data encompassing speech, text, video, and image to detect ASD-related markers with greater automation and precision^13–18^. For example: A contrastive learning framework for decision tree-based action classification leverages adjacency matrices and skeleton graphs to model periodicity and symmetry, enabling robust recognition of human interactions with potential in video-based ASD diagnosis^16^. A few-shot learning framework leveraging facial dynamics and scene-level fusion analyzes hour-long ADOS videos, classifying individuals into Autism, Autism Spectrum, and Non-Spectrum with 91.72% accuracy while highlighting the value of specific interview scenes^18^. While ML approaches have shown promise, they face significant challenges: (1) High data requirements: Training effective ML models requires large, labeled datasets. However, collecting such data is expensive, as it involves long, structured interactions between examiners and patients^19^. (2) Difficulties in extracting distinctive language disorder patterns: Traditional ML methods often struggle to reliably identify and characterize subtle yet diagnostically critical language disorder patterns, such as echolalia, pronoun reversal, or atypical pragmatic usage. These linguistic markers frequently vary across contexts and individuals, complicating their automated extraction and limiting the precision and clinical utility of ML-based approaches.

Large language models (LLMs), including GPT-3.5 and GPT-4o developed by OpenAI^20^, have shown general proficiency in language understanding tasks across a range of domains. Their application in the context of ASD assessment, however, remains largely exploratory. Unlike conventional machine learning models that typically require extensive labeled datasets, LLMs are trained on large-scale text corpora and can perform reasonably well in low-resource settings. This characteristic makes them potentially useful for analyzing ASD-related communication behaviors, especially when labeled clinical data are scarce. In this study, we examine the extent to which these models can assist in identifying linguistic features associated with ASD, with the understanding that they are not diagnostic tools but may serve as supplementary aids to clinical judgment. Despite their potential, applying LLMs in ASD diagnosis remains an emerging field with the following motivations:

- *Advancing diagnostic sensitivity and efficiency*. Diagnosing autism-related language disorders is challenging due to subtle and variable symptoms. This study leverages LLMs to achieve substantial improvements in sensitivity (recall), ensuring better detection of language deficits while streamlining the diagnostic process for timely interventions.
- *Identification of specific linguistic features*. Current methods for identifying language disorders in autistic individuals often rely on broad assessments that miss specific features. LLMs pattern recognition refines this process by identifying key linguistic markers like echolalia, pronoun reversal, and atypical language use. These insights are crucial for developing personalized treatment plans.
- *Enabling personalized therapeutic strategies*. By combining high sensitivity and precision, LLMs facilitate targeted therapeutic approaches for ASD, addressing individual patient needs. This framework also opens avenues for extending AI-driven diagnostic tools to other cognitive and developmental disorders.

**Fig. 1:**
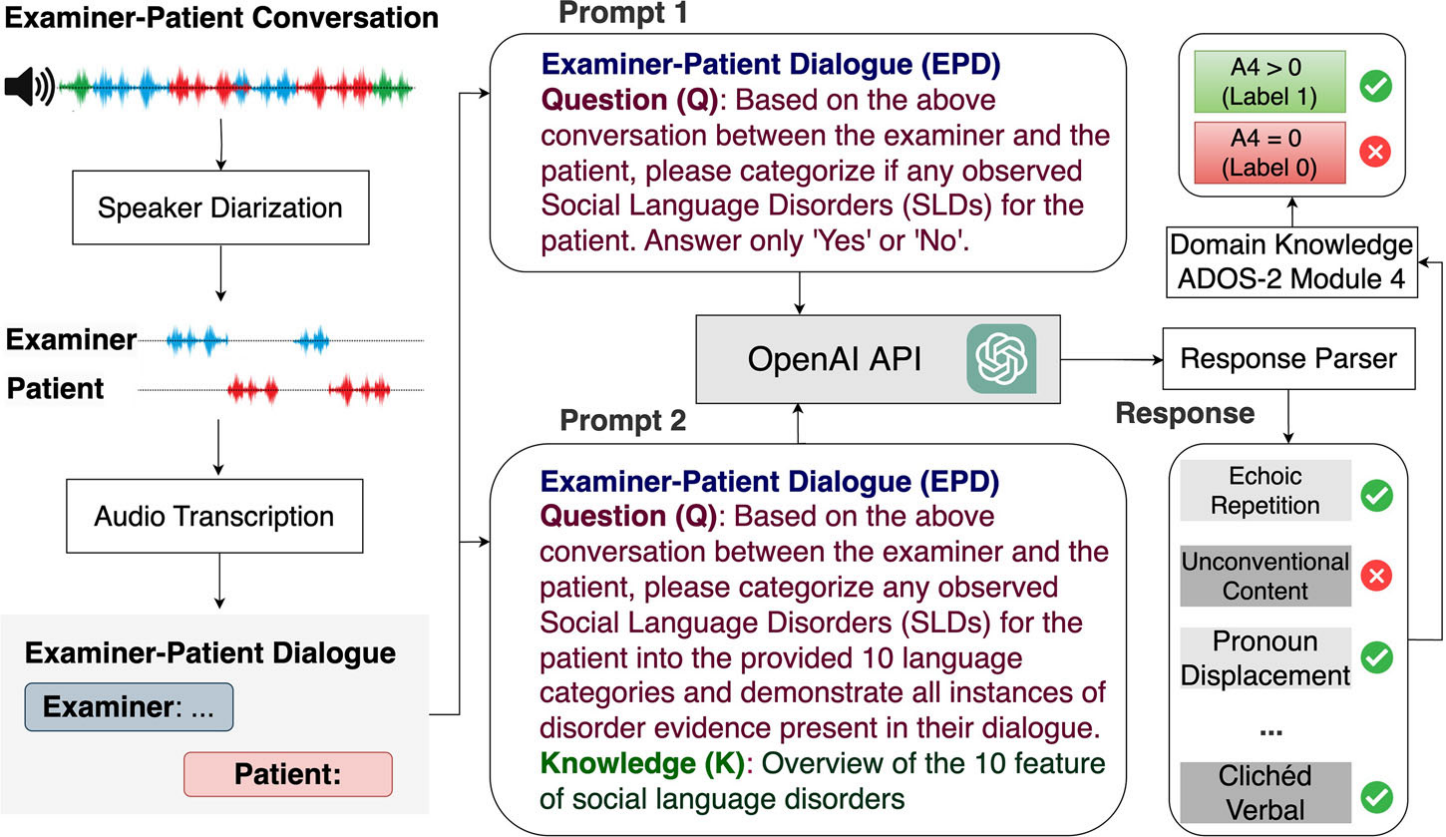
The overall framework for diagnosing autism and identifying language disorders. The conversation is first passed to stages of speaker diarization and audio transcription. Then we designed two prompts for GPT-based Models to generate a response parser. The generated response parser was mapped to an A4 Score (based on domain knowledge from ADOS-2 Module 4): Stereotyped/Idiosyncratic Use of Words or Phrases (see Table 3).

Building on the limitations of traditional diagnostic tools, this study leverages the language processing capabilities of LLMs to address these challenges. By comparing its performance with existing supervised learning models, we aim to highlight the potential of LLMs in identifying language deficits associated with ASD. This research integrates LLM technologies with domain-specific knowledge to enhance diagnostic efficiency while identifying distinct linguistic features indicative of ASD (Fig. 1). The following sections outline the datasets, experimental setup, and model comparisons to substantiate these claims.

## Results

### Experimental dataset

The ADOS-2^21,22^ is an update and extension of the original ADOS, which is a standardized diagnostic tool for ASD. The ADOS assesses communication, social interaction, play, and restricted and repetitive behaviors. It provides a series of structured and semi-structured tasks that involve social interactions between the examiner and the person being assessed. Module 4 of the ADOS-2 is designed for verbally fluent adolescents and adults (see Table 1 for description of tasks). In addition, Module 4 of the ADOS-2 organizes observations into five main areas, assessing various aspects of interaction and communication critical for diagnosing ASD in verbally fluent adolescents and adults. Table 2 provides a summary of these categories, including the specific items they encompass and their respective descriptions: each participating in 15 different scenarios (see Table 1) designed to elicit communicative and social responses that are indicators of ASD. The scenarios were structured to cover a comprehensive range of social interactions and communicative behaviors.

**Table 1:** Overview of scenario tasks in ADOS-2 Module 4 diagnosing process

Scenario	Name	Explanation
*S*_1_	Construction task	Involves the participant engaging in a task that requires constructing or assembling a set structure, testing spatial and motor skills, rather than communicative abilities.
*S*_2_	Telling a story from a book	Primarily a monologic task where the participant recounts a story from a book, differing from spontaneous dialogic interactions.
*S*_3_	Description of a picture	Participants describe a picture, testing their ability to interpret visual information and articulate a coherent description.
*S*_4_	Conversation and reporting	Focuses on the ability to engage in back-and-forth conversation and to report on past events.
*S*_5_	Current work and school	Discusses participants’ current educational and occupational engagements.
*S*_6_	Social difficulties and annoyance	Elicits experiences of social challenges and annoyances.
*S*_7_	Emotions	Requires participants to express and identify emotions.
*S*_8_	Demonstration task	Requires the participant to demonstrate how to use an item or explain a process, which does not involve interactive communication with an examiner.
*S*_9_	Cartoons	Involves interpreting sequences and explaining cartoon strips.
*S*_10_	Break	A pause or intermission in the assessment, involving no communicative or cognitive tasks.
*S*_11_	Daily living	Covers daily routines and personal care tasks.
*S*_12_	Friends, relationships, and marriage	Discusses personal relationships and social norms regarding friendships and marital status.
*S*_13_	Loneliness	Addresses feelings and situations of loneliness and isolation.
*S*_14_	Plans and hopes	Involves discussing future aspirations and plans.
*S*_15_	Creating a story	Tests creative storytelling abilities in an unstructured task.

**Table 2:** Detailed assessment categories for the ADOS-2 Module 4 observations

Class	Name	Items	Description
A	Language and communication	A1 - A10	Assesses the ability to use speech and gestures in communication effectively, evaluating the clarity, coherence, and appropriateness of language used in social interactions.
B	Reciprocal social interaction	B1 - B13	Focuses on non-verbal and verbal behaviors used in social interactions, including eye contact, facial expressions, body postures, and the quality of speech interactions.
C	Imagination/Creativity	C1	Evaluates the subject’s ability to use imagination and creativity in their expressions and thoughts, such as storytelling or creating novel responses to social scenarios.
D	Stereotyped behaviors and restricted interests	D1 - D5	Includes specific behaviors that are repetitive, restricted, and stereotyped. This category assesses the frequency and intensity of these behaviors as indicators of ASD.
E	Other abnormal behaviors	E1 - E3	Observes behaviors that are typically considered abnormal, such as overactivity, anxiety, and emotional responses that are inconsistent with the normative context.

In language-based diagnostics, the **A4 score**—part of the Stereotyped Behaviors and Restricted Interests category— becomes particularly relevant. A4 score can be evaluated using the mechanism described in Table 3: The A4 score assesses the use of stereotyped language, which is a critical indicator of ASD. A higher A4 score suggests a more frequent use of stereotyped or idiosyncratic speech, aiding in the diagnosis of ASD with higher specificity and sensitivity.

**Table 3:** A4 Score: stereotyped/idiosyncratic use of words or phrases

Score	Description
0	Rarely or never uses stereotyped or idiosyncratic words or phrases. The subject demonstrates typical language use without noticeable patterns of repetition or unusual phrasing.
1	Uses words or phrases that are more repetitive or formal compared to most individuals at a similar level of expressive language, though not obviously odd. This category also includes occasional stereotyped utterances or odd use of words or phrases, while still showing substantial spontaneous and flexible language use.
2	Often uses stereotyped utterances or odd words or phrases, with some other spontaneous language. The subject displays a noticeable pattern of repetitive or unusual phrasing that stands out in conversation.
3	Frequently uses odd or stereotyped speech and rarely exhibits non-stereotyped spontaneous speech. The language is predominantly characterized by repetitive, formal, or idiosyncratic expressions, with very little flexibility or spontaneity.

Based on the domain knowledge of professional ADOS-2 examiners, ten specific features of language deficits related to ASD have been identified. These features reflect various unconventional use patterns of language that can signify underlying social communication issues. Table 4 is a detailed description of these features:

**Table 4:** Descriptive analysis of unconventional language disorder patterns

F	Name	Explanation
*F*_1_	Echoic repetition	The individual mimics verbatim what has been said by others, including the examiner, or recites phrases from external sources like advertisements or movie scripts, showing a delayed echo response.
*F*_2_	Unconventional content	The speech contains peculiarly chosen content or contextually odd phrasing, such as using “unfreshness through household” for lack of novelty, “mideast” instead of “midwest” for U.S. states, or describing entry into a building as “through various apertures”.
*F*_3_	Pronoun displacement	Incorrectly substitutes personal pronouns, using “you” in place of “I”, or refers to themselves in the third person, either by pronouns like ’he/she’ or by their own name.
*F*_4_	Incongruous humor timing	Incorporates humorous or comedic expressions inappropriately during discussions meant to be serious, showing a misalignment between the content’s emotional tone and the context.
*F*_5_	Formalistic language use	Employs an overly formal or archaic language style that seems lifted from written texts, legal documents, or old literature, rather than engaging in conversational speech. Examples include elaborate ways of expressing simple ideas or feelings.
*F*_6_	Superfluous phrase attachment	Attaches redundant phrases or filler expressions to their speech without contributing any substantive meaning or context, such as “you know what I mean” or “as they say,” indicating a habit rather than intentional emphasis.
*F*_7_	Excessive social phrasing	Utilizes conventional social expressions excessively or inappropriately, responding with phrases like ’oh, thank you’ in contexts where it does not fit or preempting social gestures not yet performed by the interlocutor.
*F*_8_	Monotone social expression	Reiterates social phrases with an unchanged, monotonous intonation, indicating a lack of genuine emotional engagement or variability in social interactions.
*F*_9_	Stereotyped media quoting	Quotes lines from commercials, movies, or TV shows in a highly stereotypical manner, employing a canned intonation that mimics the original source closely, suggesting a reliance on external media for verbal expressions.
*F*_10_	Clichéd verbal substitutions	Resorts to well-known sayings or clichés in lieu of engaging in direct conversational responses, using phrases like ’circle of life’ or ’ready to roll’ as stand-ins for more personalized communication.

**Fig. 2:**
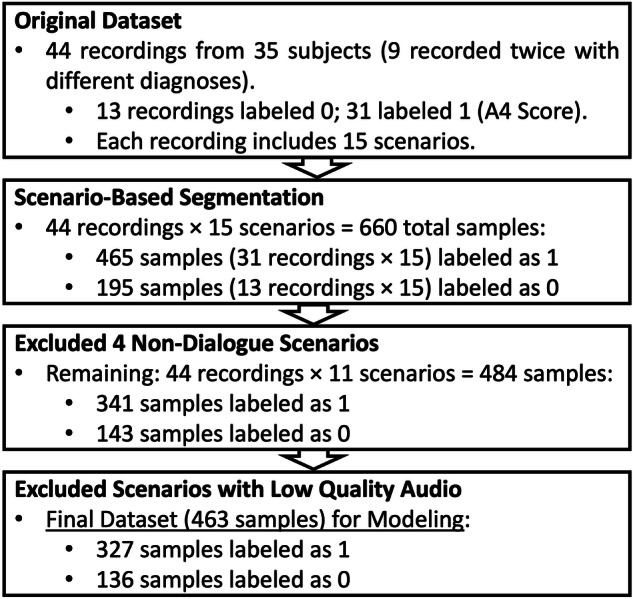
Dataset preprocessing based on ASD domain knowledge. This diagram contains the workflow of dataset preprocessing in scenario-level dialogue-based modeling.

For the purpose of this study, we utilized the Caltech ADOS Dataset^18^, which was collected during diagnostic ADOS-2 interviews (Module 4) and contains both video and audio recordings. In the paper^18^, the dataset’s primary focus was on extracting visual information from videos, such as facial dynamics, to analyze social and communication behaviors. However, our study exclusively utilizes the audio recordings from this dataset. These audio recordings are from diagnostic interviews conducted under the ADOS-2, Module 4. The Caltech ADOS Dataset involved 35 participants, aged 16 to 37 years (mean age:24.32), with a composition of 26 males and 9 females. A subset of 9 participants underwent two ADOS interviews approximately 6 months apart, resulting in a total of 44 audio recordings spanning 3316 min. Among these, 13 audio recordings have an A4 score of 0, 26 have an A4 score of 1, 5 have an A4 score of 2, and none have an A4 score of 3. Each recording was segmented into 15 sub-videos based on scene timestamps, producing 660 sub-videos with an average duration of 334 s. All were diagnosed with ASD based on the ADOS-2, confirmed through expert clinical evaluation. An overview of the data preprocessing and scenario selection workflow is illustrated in Fig. 2. In addition, each audio was involved in 15 different scenarios that were designed to elicit social and communicative behaviors characteristic of individuals on the autism spectrum. Our research focused exclusively on scenarios involving direct dialogue between the examiner and the patient, as this interaction is critical for analyzing language disorders in autism. Therefore, we excluded scenarios like the *S*_1_ Construction Task and *S*_8_ Demonstration Task, which involve non-verbal or explanatory tasks and lack the necessary dialogue. The *S*_2_ Tell a Story scenario was also excluded because it involves monologic speech without interactive exchange. Additionally, the *S*_10_ Break scenario does not include any relevant communicative or cognitive tasks. These exclusions ensured that our analysis remained focused on interactions that are directly relevant to diagnosing language-related symptoms in ASD. Out of these scenarios, we specifically selected 11 that focus on social language interactions between the examiner and the patient. We also excluded some low-quality audio from our dataset. This resulted in a final dataset of 463 scenario-level samples, with 327 labeled as Category 1 (A4 score > 0) and 136 labeled as Category 0 (A4 score = 0), which were used to evaluate the performance of our proposed framework. These selected scenarios provide a concentrated dataset to analyze social communicative exchanges, crucial for identifying language-related symptoms of ASD.

Among the selected scenarios, particular emphasis was placed on analyzing the linguistic feature of “Stereotyped/Idiosyncratic Use of Words or Phrases,” which is central to understanding language disorders that may or may not be present in individuals with autism. A4 score details are presented in Table 3. In our dataset, 13 subjects had a score of 0, indicating minimal or no use of stereotyped language, while 27 had a score of 1, showing mild repetitive or formal use, and four had a score of 2, indicating frequent use of stereotyped or odd phrases. To simplify our binary classification task, we combined subjects with scores 1 and 2 into a single category (Category 1) and grouped those with a score of 0 into Category 0. This approach was necessary due to the limited number of subjects with more severe symptoms, as our sample primarily consists of adults with less pronounced symptoms. We also recognize the suggestion to include a more diverse set of dialogues, such as those from non-autistic populations, in future analyses to broaden the perspective on language use and disorders.

### Experimental setup

The experimental evaluation was conducted on the Caltech ADOS Dataset^18^, as described in the section on experimental dataset (see Fig. 1). This dataset comprises audio recordings segmented into dialogue scenarios for analysis. To provide a comprehensive comparison with our Google-based speaker diarization approach, we evaluated two baseline models: Microsoft Azure Speaker Diarization and Pyannote speaker-diarization-3.1^23^. The Microsoft Azure system was configured with SpeakerDiarizationWithTranscription mode, a maximum of 2 speakers, and output in detailed mode, providing timestamps, speaker attribution, and confidence scores. The input audio files were preprocessed to single-channel WAV format with a sampling rate of 16 kHz. In contrast, Pyannote’s speaker-diarization-3.1 pipeline, leveraging pre-trained Pyannote/segmentation-3.0 and clustering algorithms, was configured with a minimum of 2 speakers and a maximum of 5 speakers, along with tuned overlap handling and segmentation thresholds. Both baseline models served to benchmark the diarization accuracy and robustness of our Google-based implementation, highlighting differences in handling overlapping speech and speaker variability.

The baseline models used in this study include XLNet, ALBERT, DistilBERT, RoBERTa, and BERT. For consistency across models, we adopted the following common hyperparameters: a batch size of 16, a learning rate of 3 × 10^−5^ using the Adam with Weight Decay (AdamW) optimizer^24^, a weight decay of 0.01, and a maximum sequence length of 512 tokens. A dropout rate of 0.1 was applied throughout. Model-specific configurations included the pre-trained models (xlnet-base-cased, albert-base-v2, distilbert-base-uncased, roberta-base, and bert-base-uncased). The hidden size for most models was 768, except for DistilBERT, which uses a reduced number of layers (6 instead of 12) for efficiency. All models had 12 attention heads and layer-wise transformer depths of 12, except for DistilBERT, which retains the compact architecture of its original design. To extend our evaluation beyond GPT-based models and improve the robustness of our findings, we additionally incorporated Gemini Flash 2.0, Mistral, and Qwen2. Like GPT-3.5 and GPT-4o, these LLMs were evaluated in a zero-shot setting using the same set of structured diagnostic prompts and input transcripts. This addition allows for a broader comparison across LLM families and helps assess the consistency of language-based ASD predictions beyond a single provider.

Given the relatively small size of the dataset, we designed our evaluation strategy to maximize the use of all available data while ensuring reliable and unbiased model assessment. The dataset was divided into five equally sized subsets, and we employed a leave-one-subset-out strategy. For each iteration, one subset was used as the test set (20% of the data), while the remaining four subsets (80% of the data) were used for training. This process was repeated five times, and each subset was used as the test set exactly once. At the end of the process, predictions from all iterations were combined to calculate the overall performance metrics. This approach was specifically chosen for two key reasons: (1) Small dataset: The limited size of the dataset necessitated maximizing the use of all data for both training and evaluation to obtain robust and generalizable performance metrics; (2) Fair comparison with LLMs: To compare supervised learning models with zero-shot LLMs, which generate predictions on the entire dataset in a single pass, it was critical to ensure that predictions from supervised models also covered the full dataset. This evaluation strategy ensures that every sample contributes to the assessment while avoiding data leakage between training and testing.

The effectiveness of the GPT model was evaluated using several metrics: Accuracy: The proportion of total diagnoses that were correctly identified by the model. Positive predictive value (PPV): The ratio of correct positive observations to the total predicted positives. sensitivity: The ratio of correct positive observations to the actual positives in the data. F1 Score: The harmonic mean of PPV and sensitivity, providing a single metric to assess the balance between PPV and sensitivity.

All baseline models were trained on a workstation equipped with two NVIDIA 3090 Ti GPUs, providing a total of 48 GB of VRAM. This setup ensured sufficient computational resources for processing datasets and fine-tuning the models efficiently. The hardware allowed for accelerated training times and facilitated the handling of memory-intensive tasks such as tokenization and batch processing, which were critical for achieving consistent results across all models.

### Comparison of language deficit diagnosis

Due to data limitations, cases with an A4 score of 3, representing extremely severe conditions, are not included in the dataset, and cases with an A4 score of 2 are extremely rare (42 audio recordings, 14 A4 = 0, 23 A4 = 1, 5 A4 = 2, and 0 A4 = 3). Therefore, we categorized A4 > 0 as Label = 1 and A4 = 0 as Label = 0 to explore whether large models can assist in diagnosing patients and identifying the presence of social language disorders. To compare the performance of different GPT versions, our backbone models were evaluated based on the API versions GPT-3.5-turbo and GPT-4. Table 5 summarizes the results of this comparison.

**Table 5:** Performance metrics of different models

Model	Accuracy	PPV	Sensitivity	F1 score
XLNet^30^	58.76%	54.38%	58.76%	56.07%
ALBERT^31^	69.07%	47.71%	69.07%	56.44%
DistilBERT^32^	58.76%	51.85%	58.76%	54.44%
RoBERTa^33^	57.73%	58.12%	57.73%	57.92%
BERT^34^	63.92%	60.86%	63.92%	61.87%
Mistral - 7b	70.29%	70.29%	100.00%	82.55%
Qwen2 - 7b	70.29%	70.29%	100.00%	82.55%
Gemini 2.0 Flash-based	63.47%	72.34%	77.97%	75.05%
GPT 4o - based	67.05%	70.76%	90.51%	79.43%
GPT 3.5 - based	69.14%	71.52%	93.22%	80.94%

Table 5 demonstrates that LLMs achieved better performance across all metrics, outperforming the baseline models such as BERT, RoBERTa, and XLNet. Notably, the performance of the two GPT versions was remarkably similar, indicating the consistency and robustness of the GPT-based approach for diagnosing social language disorders (SLDs). In contrast, the baseline models lagged in all metrics, highlighting the advantage of using LLMs for this task.

To further examine the impact of speaker diarization tools and underlying LLMs, we conducted ablation experiments that varied (1) the speaker diarization pipeline and (2) the LLM used for zero-shot diagnosis. Specifically, we tested combinations of GPT-based models and Gemini Flash 1.5, with multiple diarization strategies, including Pyannote, Microsoft Azure, Google Diarization API, and human-annotated reference labels.

Integrating Google’s speaker diarization (SD) technology with our GPT-based model significantly improved all performance metrics, achieving the highest scores overall. Unlike other diarization tools, Google SD not only distinguishes multiple speakers (e.g., Speaker 1, Speaker 2) but crucially identifies whether the speaker is the examiner or the patient. This capability is especially valuable for tasks requiring an understanding of interaction dynamics and speaker roles (examiner vs. patient), which significantly influence the model’s performance in contextual analysis and response generation.

Among LLMs, GPT-based models achieved the highest overall performance, particularly in terms of accuracy (82.00%), positive predictive value (91.06%), and F1 score (86.57%). The Gemini Flash 1.5 model also performed strongly in the same configuration, achieving comparable sensitivity (89.63%) and a competitive F1 score (80.35%), but with slightly lower accuracy and PPV than GPT-3.5.

These results indicate that while both LLM families are capable of supporting zero-shot diagnostic inference when paired with accurate diarization, GPT-based models currently show slightly more stable performance across evaluation metrics in our task setting. Furthermore, integrating speaker-role-aware diarization, especially human-labeled or high-quality automated pipelines, proves essential in unlocking the full diagnostic potential of LLMs for language-based ASD assessment.

### Analysis of features of language deficit

**Fig. 3:**
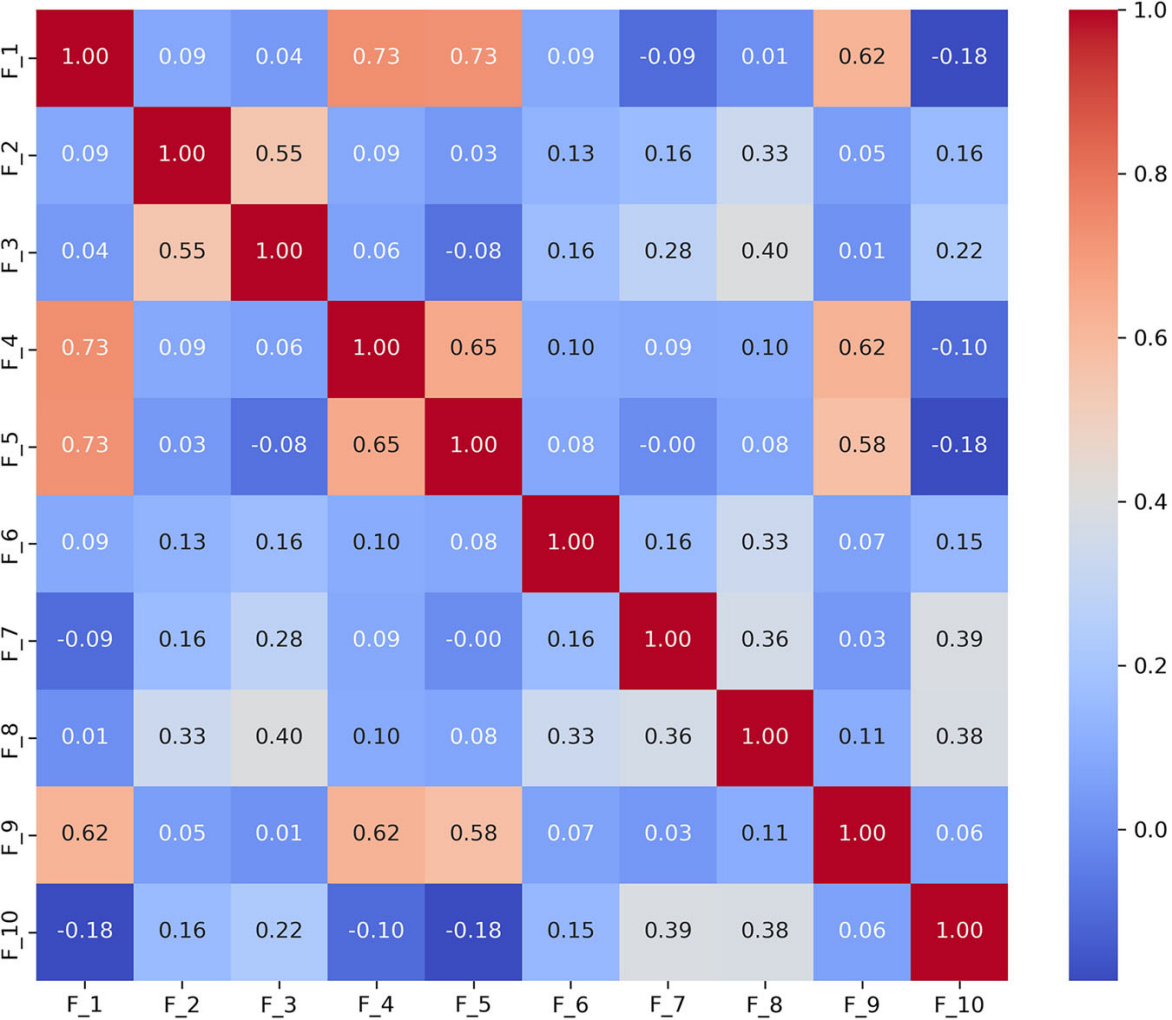
Correlation coefficients between features of language deficit. The correlation between features of language deficit in ASD offers valuable insights into the complex nature of communication challenges faced by individuals on the spectrum. In this study, we have found that the correlation pairs (*F*_1_, *F*_4_), (*F*_1_, *F*_5_), (*F*_1_, *F*_9_), (*F*_2_, *F*_3_), (*F*_4_, *F*_5_), (*F*_4_, *F*_9_), (*F*_5_, *F*_9_) are noticeably higher than the remaining pairs including five negative pairs (*F*_1_, *F*_10_), (*F*_1_, *F*_7_), (*F*_3_, *F*_5_), (*F*_4_, *F*_10_), (*F*_5_, *F*_10_). Together, the correlation between features of language deficit in ASD offers valuable insights into the complex nature of communication challenges faced by individuals on the spectrum.

To thoroughly understand the interrelationships between different language features identified in the ASD diagnostic assessments, we analyzed the interrelationships among ten language features (*F*_1_ to *F*_10_) derived from the dataset. These features represent various aspects of language use that may indicate ASD, such as repetitive use of words or unusual language patterns. We calculated Pearson correlation coefficients between each pair of features to quantify their linear relationships. Each language feature is represented as a binary variable, where “1” indicates the presence and “0” indicates the absence of that specific language disorder feature within any given sample. For example, if a feature detected by GPT, such as “echolalic repetition”, is observed in the dialogue during a diagnostic session, it is marked as “1” for that session; otherwise, it is marked as “0”. This binary coding allows us to apply Pearson correlation to measure the linear relationship between each pair of features across all samples. This analysis pinpoints which features often co-occur within the linguistic profiles of ASD diagnosed through ADOS-2, Module 4. The computed correlation matrix for the features is presented in Fig. 3.

We have made the following observations: (1) Highly Correlated Features: a) *F*_1_, *F*_4_, and *F*_5_: These features show very high correlations (*r* = 0.734 between *F*_1_ and *F*_4_, *r* = 0.727 between *F*_1_ and *F*_5_, and *r* = 0.655 between *F*_4_ and *F*_5_). This suggests they may capture similar aspects of linguistic behavior, possibly related to the repetitive or stereotyped use of language, which is a common indicator of ASD. b) *F*_4_ and *F*_9_: Another pair, *F*_4_ and *F*_9_ (*r* = 0.622), indicates a strong association, which might reflect overlapping features of language presentation in ASD, such as idiosyncratic language use or atypical language processing. (2) Moderately Correlated Features: *F*_2_ and *F*_3_: These features exhibit moderate correlations (*r* = 0.55 for F2 and F3), with F2 and F3 showing a correlation strength that is typically described as “moderate” according to established guidelines for interpreting correlation coefficients. These correlations suggest a meaningful but not strong relationship in linguistic traits, such as variability in speech that includes both repetitive and novel elements. (3) Negatively Correlated Features: *F*_1_ and *F*_10_: The negative correlation (*r* = −0.184) suggests that when *F*_1_ (possibly denoting less severe ASD indicators) is present, *F*_10_ (perhaps denoting more severe ASD indicators) is less likely to be present, and vice versa. This can help differentiate levels of language impairment in ASD diagnoses. Together, the correlation between features of language deficit in ASD offers valuable insights into the complex nature of communication challenges faced by individuals on the spectrum.

This subsection analyzes the correlations between linguistic features across various ADOS scenarios to identify patterns that may indicate language disorders associated with ASD. The focus is on scenarios with direct dialogue between the examiner and the patient, reflecting our study’s emphasis on communication. While Scenarios *S*_1_, *S*_2_, *S*_8_, and *S*_10_ provide valuable insights into various aspects of cognitive and social functioning, they were not included in this analysis due to their lack of direct dialogue-based interaction between the examiner and the patient, which is a primary focus of our research. Table 6 To effectively analyze the differences in the distribution of values from *F*_1_ to *F*_10_ across various scenarios, we conducted a detailed statistical examination (see Table 7). This analysis helps to understand how the prevalence of each linguistic feature associated with ASD varies across the scenarios, which can provide insights into the contexts or conditions under which certain features are more likely to appear.

**Table 6:** Performance comparison across different approaches

Model	Accuracy	PPV	Sensitivity	F1 score
w/o speaker diarization	63.64%	48.12%	63.64%	54.80%
w/ pyannote^23^	68.18%	59.68%	68.18%	60.45%
w/ Microsoft^35^	72.73%	71.25%	72.73%	71.98%
w/ Google^36^	69.14%	71.52%	**93.22%**	80.94%
w/ human diarization + Mistral	56.60%	75.07%	57.68%	65.24%
w/ human diarization + Qwen	49.21%	73.51%	43.88%	54.95%
w/ human diarization + Gemini	69.10%	72.81%	89.63%	80.35%
w/ human diarization + GPT	**82.00%**	**91.06%**	82.49%	**86.57%**

Bold values represent the best performance in each column metric.

**Table 7:** Prevalence of linguistic features by scenario indicative of language deficits in ASD

Scenario	*F*_1_	*F*_2_	*F*_3_	*F*_4_	*F*_5_	*F*_6_	*F*_7_	*F*_8_	*F*_9_	*F*_10_
3	0.45	0.64	0.52	0.32	0.39	0.59	0.48	0.41	0.39	0.36
4	0.57	0.59	0.45	0.32	0.41	0.59	0.57	0.39	0.30	0.32
5	0.43	0.55	0.41	0.23	0.43	0.61	0.48	0.27	0.25	0.32
6	0.41	0.34	0.25	0.18	0.36	0.55	0.43	0.23	0.07	0.30
7	0.48	0.39	0.34	0.20	0.36	0.50	0.39	0.30	0.20	0.30
9	0.57	0.43	0.30	0.20	0.32	0.48	0.41	0.23	0.16	0.20
11	0.43	0.39	0.32	0.14	0.32	0.59	0.55	0.32	0.18	0.32
12	0.45	0.55	0.41	0.18	0.36	0.55	0.61	0.41	0.07	0.32
13	0.55	0.36	0.25	0.02	0.36	0.48	0.30	0.25	0.02	0.23
14	0.36	0.41	0.18	0.11	0.23	0.34	0.20	0.11	0.07	0.11
15	0.64	0.57	0.32	0.36	0.52	0.64	0.57	0.32	0.30	0.20

We have derived the following observations and insights: (1) Feature Prevalence: The occurrence rates of features *F*_2_, *F*_6_, and *F*_7_, which represent aspects of unconventional content, verbal fluency, and excessive social phrasing, respectively, were consistently above 60% across most scenarios. This high prevalence underscores their significance as key indicators of ASD. (2) Language Feature in Social Contexts: Features such as *F*_1_ (possibly related to echolalia or repetitive speech), *F*_4_, *F*_5_, and *F*_9_ (potentially related to atypical or stereotyped language use) were absent in several scenarios, underscoring their sensitivity to specific social or communicative contexts. (3) Scenario-Specific Patterns: High prevalence rates in *F*_7_ during the *S*_7_ (i.e., “Emotions”) scenario, and diverse responses in *F*_2_ across the *S*_12_ (i.e., “Friends, Relationships, and Marriage”) and *S*_15_ (i.e., “Creating a Story”) scenarios suggest that certain linguistic features were particularly elicited by emotional or social relational contexts.

**Fig. 4:**
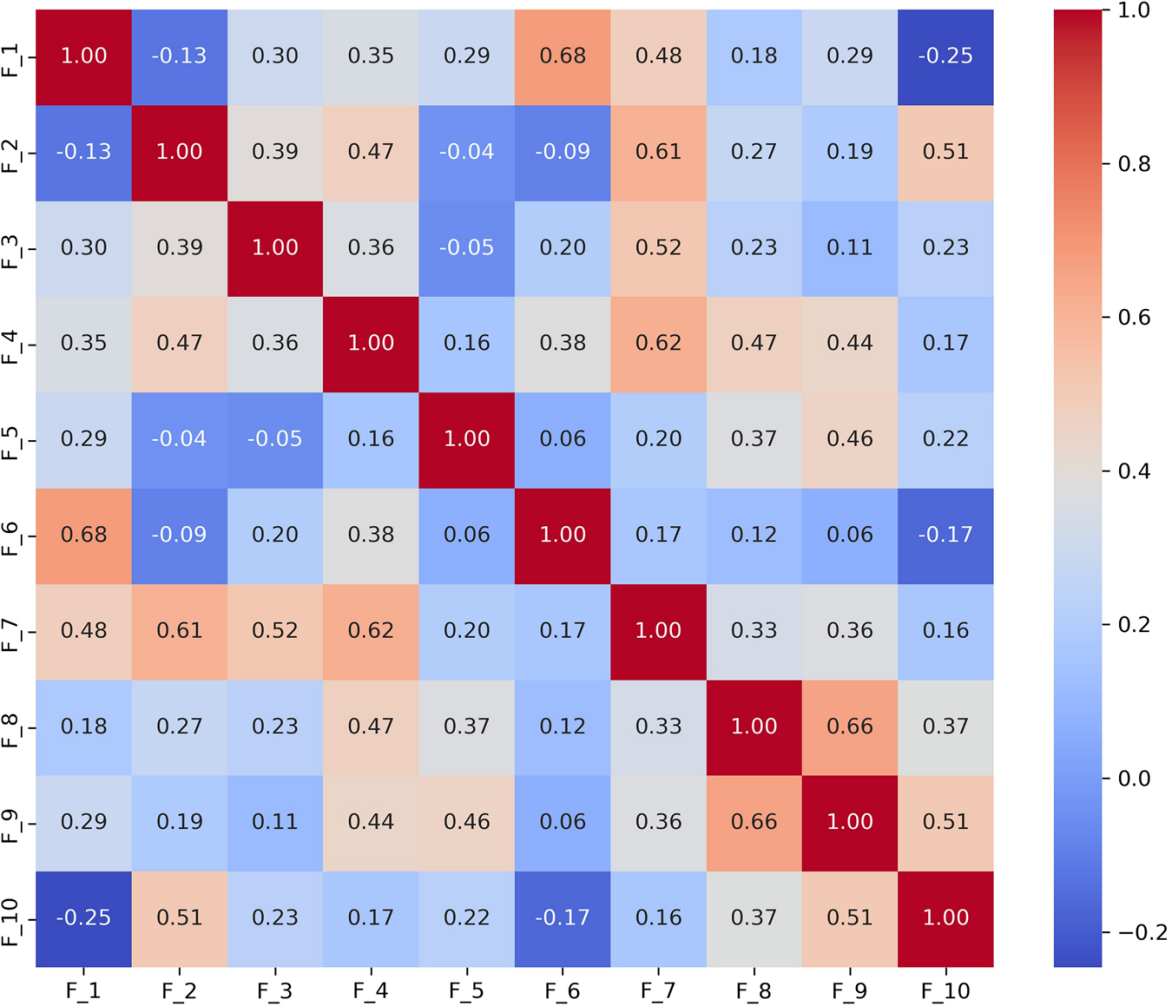
Correlation matrix of linguistic features *F*_1_ to *F*_10_ in the *S*_3_ (i.e., “Description of a Picture”) scenario. The matrix shows strong correlations between features (e.g., (*F*_1_, *F*_6_), *F*_2_, *F*_7_, and *F*_4_, *F*_7_), underscoring the interdependencies that influence how visual information is described. This pattern may indicate that individuals with ASD use scripted language as a strategy to manage social interactions, although this can often result in conversations that seem awkward or misplaced.

**Fig. 5:**
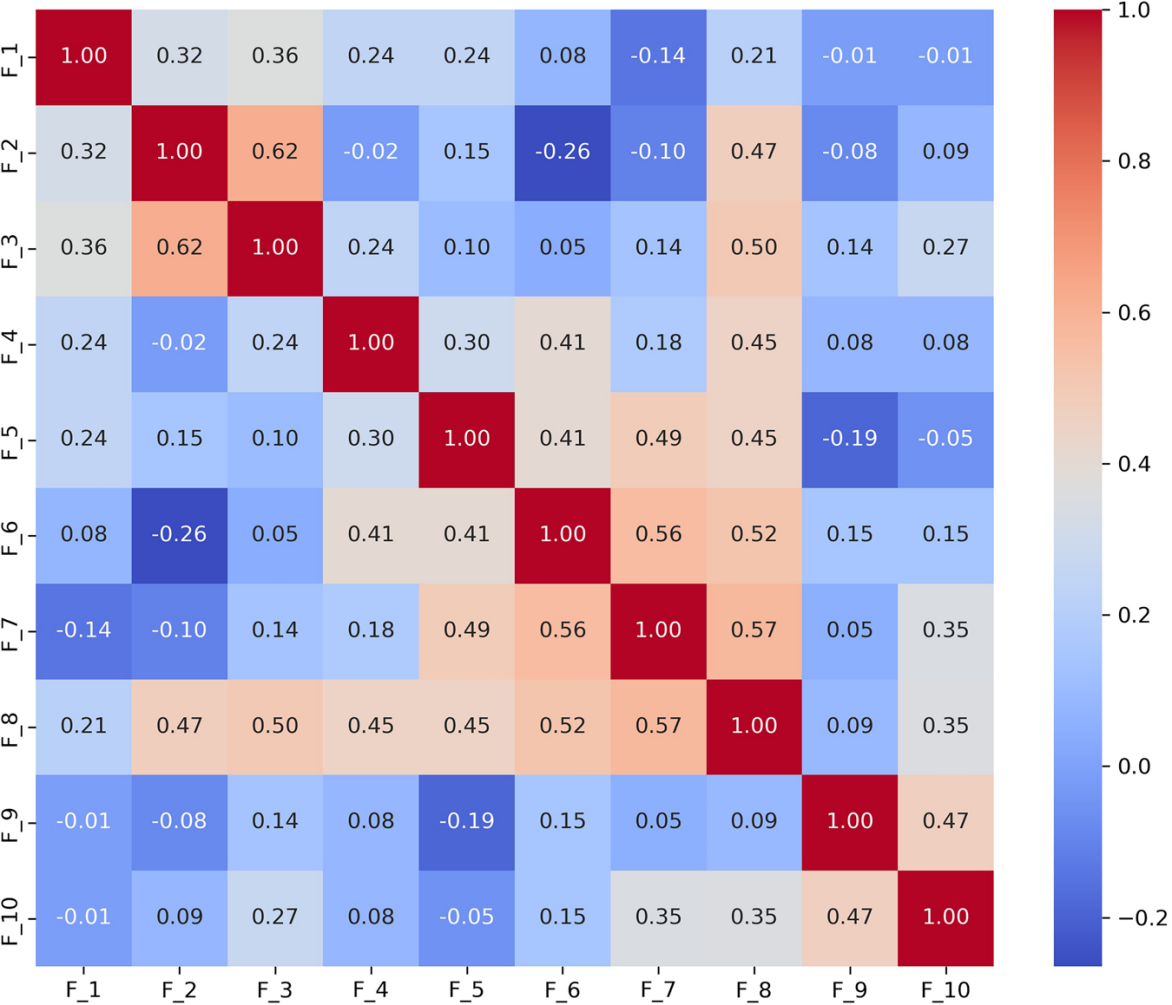
Correlation matrix of linguistic features *F*_1_ to *F*_10_ in the *S*_9_ (i.e., “Cartoons”) scenario. This matrix highlights correlations that elucidate the cognitive and perceptual challenges in interpreting cartoons (e.g., *F*_2_, *F*_3_, and *F*_7_, *F*_8_), essential for understanding narrative contexts and humor in ASD. Such speech patterns can make it difficult for them to engage in spontaneous and emotionally responsive interactions, which are critical for successful social exchanges.

To further demonstrate the utility of this analysis, we specifically focused on the *S*_3_ and *S*_9_ scenarios, which are essential for evaluating narrative skills and abstract reasoning, respectively. We derived the following results: (1) **Scenario** *S*_3_ (Fig. 4): The strong correlation between *F*_1_ and *F*_6_ (0.68) indicates challenges in effectively summarizing visual content. This may reflect difficulties in processing and conveying information succinctly, which is often a challenge for individuals with ASD. A high correlation (0.66) between *F*_8_ (monotone social expression) and *F*_9_ (stereotyped media quoting) suggests that individuals may struggle with varying their emotional expressions, which could affect the emotional richness of their speech. Correlations between *F*_7_ (excessive social phrasing) and *F*_4_ (incongruous humor) (0.62), and between *F*_7_ and *F*_2_ unconventional content (0.61), suggest a connection between repetitive social expressions and the production of either inappropriate humor or atypical content. This pattern may indicate that individuals with ASD use scripted language as a strategy to manage social interactions, although this can often result in conversations that seem awkward or misplaced. (2) **Scenario** *S*_9_ (Fig. 5): The strong correlation between *F*_2_ and *F*_3_ (0.62) suggests that individuals with ASD might struggle to adjust their language to fit the context appropriately. This is particularly problematic in scenarios like watching and discussing cartoons, where understanding shifting dialogues and multiple characters’ perspectives is essential; Additionally, *F*_8_’s significant correlations with *F*_7_ (0.57) reflect difficulties in varying emotional tone and using phrases that might be socially appropriate. Individuals exhibiting these features tend to speak in a flat, unmodulated manner while possibly overusing certain social phrases, making their speech seem rigid and scripted. Such speech patterns can make it difficult for them to engage in spontaneous and emotionally responsive interactions, which are critical for successful social exchanges.

**Fig. 6:**
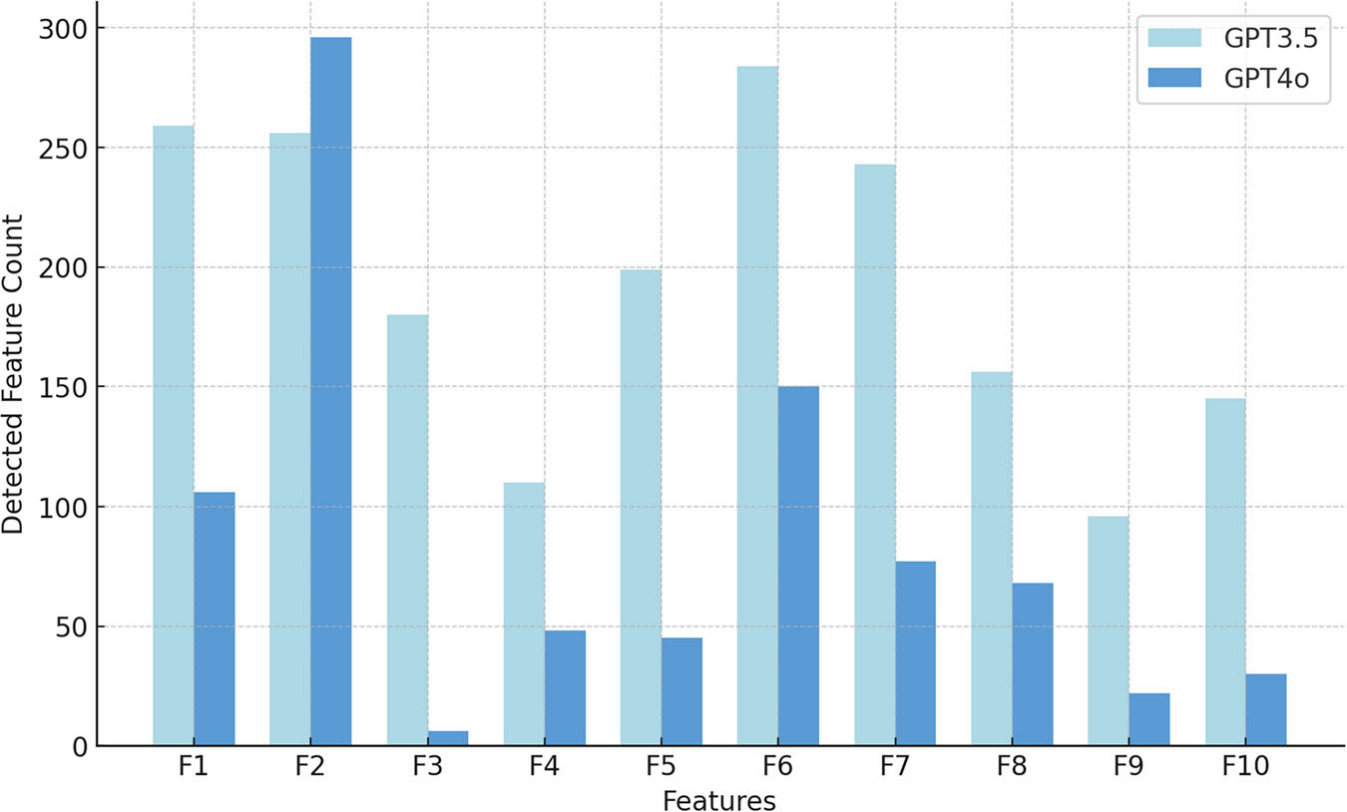
Impact of LLMs selection on feature count. We have observed the difference between GPT-3.5 and GPT-4o when detecting SLD Features F1-F10. We observe that GPT-4o adopts a more conservative approach than GPT-3.5, showing a slightly lower detection rate across key ASD-related markers.

Together, these correlations suggest co-morbid linguistic challenges that individuals with ASD may encounter in scenarios requiring detailed visual interpretation or complex narrative understanding. To investigate how different features influence model performance, we further analyze the performance of the models through a feature detection comparison. Figure 6 provides a histogram comparing the counts of specific linguistic features related to SLDs detected by GPT3.5-based and GPT4o-based models.

Overall, the performance of GPT-3.5 and GPT-4o is quite similar, with both models achieving comparable sensitivity and F1 score. However, a closer analysis reveals that GPT-3.5 tends to make more aggressive predictions, detecting a broader range of linguistic features associated with ASD, such as F1 (Echoic Repetition), F6 (Verbal Fluency), and F7 (Excessive Social Phrasing). In contrast, GPT-4o adopts a more conservative approach, identifying fewer features overall and showing a slightly lower detection rate across key ASD-related markers. While GPT-4o’s cautious predictions may reduce over-detection. These differences suggest that GPT-3.5 provides a more expansive diagnostic perspective, capturing more ASD-related speech patterns, whereas GPT-4o maintains a more selective, risk-averse approach in feature identification.

### Case study

Lastly, we present case studies to illustrate the practical application of GPT in identifying language deficits in ASD. Tables 8 and 9 present case studies that illustrate how GPT detects linguistic features indicative of social language disorder (SLD) during the prediction process. These examples highlight the conversational challenges commonly faced by individuals with ASD, showcasing specific linguistic patterns that signal underlying language impairments. By integrating GPT with structured conversational analysis, these case studies demonstrate its potential for identifying key diagnostic features of ASD-related language deficits. The extracted features closely align with established ASD communication characteristics, providing a strong foundation for further diagnostic assessment and intervention planning.

**Table 8:** Case study analysis: identifying language deficits in an examiner-patient dialogue

Examiner (E) - Patient (P) dialogue	
E: Okay. So, do you have some friends?	
**P: Uh**, do I have some ${{\bf{friends?}}}_{{{\rm{F}}}_{1}}$	
E: Um-hum.	
**P:** Well, pretty ${{\bf{much}}}_{{{\rm{F}}}_{10}}$, three of them from peers.	
E: Three of them from here?	
**P: Um-hum**.	
E: Okay. Can you tell me a little bit about them?	
**P:** Well, they’re kind of living near, they kind of live near ${\text{her}}_{{{\rm{F}}}_{3}}$ farther from here.	
E: They’re further from here? What do they like?	
**P:** What do they ${{\rm{like?}}}_{{{\rm{F}}}_{1}}$ They’re kind of energetic, just like me. Cool.	
E: Um, and what do you guys like to do together? Man, we like movies and stuff.	
**P: And you’ve got to know them through peers**.	
...	
E: And, but you said you’d go, you’d like to go to movies and stuff as well. Do you go to movies with them, or?	
**P:** We use ${\text{gas movies}}_{{{\rm{F}}}_{9}}$.	
E: Oh, you talk about it?	
**P: Yeah**.	
E: Okay. And are there people outside of peers that you’re friends with, or?	
**P: You mean, uh**, outside of ${\text{peers}}_{{{\rm{F}}}_{1}}$?	
...	
**P: Crazy, crowded, crooked. One of those years people**.	
E: Oh.	
**P: They** triggered the trip, pregnant sound ${\text{effects}}_{{{\rm{F}}}_{9}}$.	
E: Oh, yeah.	
**P: When they asked about dating. Um What, where do you, uh, want to live when you get older**	
E: face?	
**P:** I want to live in a lounge and dirty autistic ${\text{matching}}_{{{\rm{F}}}_{2}}$. You know, you can zillion blocks ${\text{matching}}_{{{\rm{F}}}_{10}}$.	
E: And who do you think you would like to live with, with your family or roommates or by yourself?	
**P: I want to live with my family there. Okay**	
...	
**Extracted features based on ChatGPT response**	
**Echoic repetition** (*F*_1_): When the examiner first asked the patient whether they have some friends, the patient echoed the question back at the examiner before answering. In subsequent interactions, the patient frequently mimics the examiner’s questions verbatim before answering.	
**Unconventional content** (*F*_2_): The patient refers to living in “a lounge and dirty autistic matching” rather than using any conventional descriptions for living spaces. Similarly, the phrase “zillion blocks matching" has an unusual content.	
**Pronoun displacement** (*F*_3_): The patient referred to his own house as “her".	
**Stereotyped media quoting** (*F*_9_): The patient quoted “gas movies” and “triggered the trip, pregnant sound effects”, which seems to be quoted from an external media source.	
**Clichéd verbal substitutions** (*F*_10_): The patient uses clichéd expressions like “well, pretty much” instead of giving direct responses.	

Phrases highlighted in blue indicate observed linguistic anomalies, while red underscores the specific feature category of language deficits.

**Table 9:** Case study analysis: identifying language deficits in an examiner-patient dialogue

Examiner (E) - Patient(P) Dialogue	
E: So, I’m going to ask you a few questions about work and school	
**P: Yes**.	
E: Um, first of all, do you have a job?	
**P: No, I used to be laid off**.	
...	
E: And that’s okay? Yeah Um, while you were working or now at school, or at high school, maybe before that, did you have a group of, any problems getting along with people You weren’t in high school?	
**P: Any school. Well, like, like**, stupid schools for you when I was developing angry or high ${\text{school.}}_{{{\rm{F}}}_{2}}$	
...	
E: What kind of things you used to bother you that other people did?	
**P:** Like, uh, when I was in the school bus, I had students grabbing my backpack, whatever, and I didn’t mad it or ${\text{suck.}}_{{{\rm{F}}}_{10}}$	
...	
E: And have you ever done anything so that other people wouldn’t teach soon?	
**P:** Yes, but sometimes they just, it’s like they’ve been doing it for a while, so it’s just kind of like Hey, you know or what, ${\text{whatever,}}_{{{\rm{F}}}_{6}}$ we’ll just tease him about something else.	
...	
**Extracted features based on ChatGPT response**	
**Unconventional content** (*F*_2_): There are instances where the patient uses unconventionally chosen phrases like “stupid schools for you when I was developing angry or high school”.	
**Superfluous phrase attachment** (*F*_6_): The patient attaches redundant phrases or filler expressions to their speech without contributing any substantive meaning or context, such as “whatever” and or “whatever”.	
**Clichéd verbal substitutions** (*F*_10_): The patient resorts to clichéd expressions when describing how he felt during certain situations: “I didn’t mad it or suck”.	

Phrases highlighted in blue indicate observed linguistic anomalies, while red underscores the specific feature category of language deficits.

To further enhance our understanding of the impact of speaker diarization on ASD diagnosis, we included a comparative analysis between human speaker diarization and Google’s automated speaker diarization tool. Table 10 presents a detailed comparison of these two approaches, highlighting instances where speaker diarization errors led to misdiagnosis.

**Table 10:** Comparison of human speaker diarization and Google speaker diarization with extracted language features

Human speaker diarization	Google speaker diarization
**E:** Okay. So you’ve mentioned a number of friends. Can you tell me about them a little bit? **P:** Well, my roommate’s name is Emily, and she’s from Minnesota, and she’s getting married this summer.	**E:** Okay. So, you mentioned a number of friends. Can you tell me? **P:** ${Um,she{\prime} sgettingmarriedthissummer.}_{{{\rm{F}}}_{2}}$
**P:** She’s been in a long-term relationship of 6 years since, like, high school. So it’s, like, pretty young, but I think she’s ready for it. **E:** Mhmm.	**P:** She’s been in a long term relationship, a six years. Since, like, high school, so, uh, it’s, like, pretty young, but I think she was ready for it. **E:** Um-hum.
**E:** That’s cool. Okay. **P:** So MRE and others. Oh, and then there’s Carrie. **E:** How do you spell it? **P:** K a r I. **E:** Okay.	**P:** Cool. **E:** Yeah. It’s going to be good. **P:** ${Okay.So,.Andothers?}_{{{\rm{F}}}_{2}}$**E:** Uh, and the miscarriage. **P:** ${KARI.}_{{{\rm{F}}}_{2}}$**E:** Okay.
**P:** We met her freshman year, in math class and then we’ve just been friends ever since.	**E:** When I’m at her freshman year, um, in math class and then we’ve just been friends ever since.
...	...
**P:** I also like having people over to my place for dinner because I like cooking and that’s fun for me and for them too. **E:** What kind of stuff do you like to cook? **P:** I don’t know. We make like enchiladas is good and also, like, different pastas but, like, with homemade sauces.	**P:** ${Wealso,like,Ithinkso,like}_{{{\rm{F}}}_{6}}$, having people over to my place for dinner. Because I like cooking. **E:** What kind of stuff do you have to cook? **P:** ${Um,kindof,meal,like,like}_{{{\rm{F}}}_{6}}$, enchiladas good, and So, like, different pastas, but, like, like homemade sauces.
**Extracted features based on GPT response**	**Unconventional content** (*F*_2_) **superfluous phrase attachment** (*F*_6_)

One notable case involves a segment from a patient labeled as 0 (indicating no social language disorder). Due to challenges such as low volume or increased distance from the microphone, Google’s speaker diarization tool misattributed parts of the examiner’s dialogue to the patient. This misattribution resulted in incorrect transcription outputs, which subsequently led to erroneous diagnostic conclusions.

Specifically, in Table 10, the patient originally did not exhibit any social language disorder features. However, due to diarization errors, their speech was altered or merged with the examiner’s, leading the system to mistakenly detect features such as *Unconventional content (F*_2_*)* and *Superfluous phrase attachment (F*_6_*)*. For example, phrases like “Okay. So, and others?” and “We also, like, I think so, like” were misclassified as signs of unconventional content and excessive verbal fillers, respectively. These distortions made the model misinterpret the patient’s speech patterns, falsely identifying them as exhibiting signs of social language disorder when, in reality, the mistakes arose from transcription errors rather than actual linguistic behavior.

The error analysis underscores the importance of accurate speaker attribution in automated ASD diagnostics. Misclassifications stemming from diarization errors can significantly skew the model’s interpretation of language features, emphasizing the need for more robust diarization techniques.

## Discussion

This study explored the feasibility and effectiveness of leveraging LLMs, specifically GPT-based models, for identifying language deficits associated with ASD. Our results demonstrate that the proposed framework exhibits promising potential in accurately detecting subtle linguistic markers indicative of SLDs, such as echolalia, pronoun reversal, and atypical pragmatic language usage. The comparative analysis showed that GPT-based models consistently outperformed traditional supervised learning models, suggesting that the language understanding capabilities embedded within LLMs can effectively generalize to complex clinical language tasks. A finding of this study was the differential performance between GPT-based models and the Google Gemini Flash model. While Gemini demonstrated comparable performance, GPT-based models slightly outperformed Gemini, particularly in terms of sensitivity and positive predictive value. This indicates that GPT-based models may have a more robust ability to detect nuanced language anomalies, though further research with larger and more diverse datasets is necessary to confirm this observation.

The integration of speaker diarization, particularly Google’s role-based system, has been pivotal in enabling the automatic diagnosis of ASD in conversational scenarios. By accurately distinguishing between examiner and patient speech, the model can better analyze patient-specific language patterns, thereby enhancing sensitivity and contextual understanding in ASD assessments. However, while automatic speaker diarization is effective, our results indicate that it still falls short compared to human diarization. In our ablation experiments, human diarization achieved the highest performance, with an Accuracy of 82.00%, PPV of 91.06%, and an F1-score of 86.57%, significantly outperforming automated methods. This underscores the crucial role of speaker diarization in ASD diagnosis and highlights the need for further advancements in diarization technology. As automated tools like Google’s system continue to evolve, their ability to match or exceed human-level precision will be essential for improving diagnostic reliability, especially in cases where conversational context and speaker role differentiation are critical.

A closer examination in Table 10 reveals that errors in Google’s speaker diarization led to misclassification of social language disorder features. In cases where patients spoke at a lower volume or were positioned farther from the microphone, the diarization system misattributed parts of their speech to the examiner. This misattribution resulted in incorrect transcription outputs, which in turn caused erroneous classification of unconventional content (*F*_2_) and superfluous phrase attachment (*F*_6_). These errors highlight the limitations of current automated diarization systems in preserving conversational integrity, emphasizing the need for improved speech recognition models and diarization algorithms to ensure more accurate ASD diagnosis.

The prevalence of language deficits across different scenarios revealed valuable patterns about how individuals with ASD respond to various social and cognitive contexts: (1) Emotional and Creative Contexts: Scenarios such as *S*_7_ (Emotions) and *S*_15_ (Creating a Story) elicited higher occurrences of features like *F*6 (verbal fluency) and *F*_7_ (excessive social phrasing). This suggests that emotional and narrative-based interactions highlight specific communication challenges in individuals with ASD, such as difficulty adapting language to dynamic social contexts or producing coherent narratives^25^. (2) Visual Interpretation Challenges: In *S*_3_ (Description of a Picture), strong correlations between *F*_1_ (repetition) and *F*_6_ (verbal fluency) indicate struggles with organizing and summarizing visual information. These patterns align with known cognitive challenges in ASD, such as difficulties in abstraction and the integration of sensory input into coherent verbal descriptions^26^. (3) Abstract Reasoning in Narratives: The results from *S*_9_ (Cartoons) highlight co-occurring features like *F*_2_ (unconventional content) and *F*_3_ (pronoun displacement), suggesting that interpreting multi-character narratives and shifting perspectives poses significant difficulties for individuals with ASD. These findings reinforce the need for scenario-specific evaluations that consider how different contexts elicit unique linguistic deficits.

The results from the present study have strong clinical implications. Our model’s ability to analyze complex language interactions is valuable in clinical settings. It allows for an earlier and more accurate detection of language deficits, which are often indicative of ASD. This early diagnosis is crucial for the timely intervention that can lead to better management and outcomes for adults with ASD. A promising future direction is to leverage the power of LLMs to distinguish ASD from other language impairments during development^27^. If LLMs can shed new insight into the co-occurrence of ASD and language impairment, clinical diagnosis of developmental disorders might benefit from human-AI collaboration.

Despite its innovations, this study has limitations that should be addressed in future research. One limitation is the relatively small dataset size, while sufficient for exploratory analyses, restricts the statistical power and generalizability of our findings. As medical data is often difficult to obtain due to privacy and accessibility constraints, future work will prioritize acquiring larger and more diverse datasets to enhance the robustness and applicability of our approach. Additionally, the effectiveness of the GPT model depends heavily on the quality and variety of the training data. The current dataset represents a relatively homogeneous population in terms of linguistic and cultural backgrounds. Expanding the dataset to include a more diverse population could improve the model’s robustness and generalizability.

Looking forward, the study highlights the potential for these technologies to incorporate a wider variety of data and to develop adaptive learning models that continually improve in accuracy and effectiveness. This progression promises to revolutionize ASD diagnostics, paving the way for more personalized and accessible care for individuals with ASD. The integration of LLMs like GPT in clinical settings is a forward step in making ASD diagnostics not only quicker and more accurate but also more comprehensive in understanding and addressing the diverse needs of the autism community. Furthermore, future studies could explore the integration of multimodal data analysis^28^ to enhance diagnostic capabilities further. Combining speech with visual cues such as facial expressions and body language could provide a more comprehensive view of an individual’s communicative and social behaviors. Finally, refining the models to incorporate feedback loops that enable continual learning from new data could adaptively improve diagnostic accuracy over time^29^. These enhancements will pave the way for more robust and comprehensive diagnostic tools in the future.

## Methods

### Framework for diagnosing autism and identifying language disorders

We analyzed videos recorded during the ADOS interview. These videos feature structured yet natural dialogues between an interviewer and the participant, capturing various behaviors indicative of ASD in adults. Written informed consent was obtained from all participants, following the ethical guidelines approved by the Institutional Review Boards (IRB) at West Virginia University (1602015509 and 1905567600R001), Washington University in St. Louis (WashU), University at Albany (UAlbany), and the California Institute of Technology (Caltech).

Building on the foundational practices established by the ADOS-2, specifically Module 4, designed for verbally fluent adolescents and adults, we have developed a comprehensive framework (see Fig. 1) that incorporates LLMs like GPT. This framework is tailored to enhance the diagnostic PPV and identification of language disorders in individuals suspected of having ASD. Specifically, it involves the following components: (1) *Speaker diarization and audio transcription*. This technology segments the audio recordings to precisely separate the speech of the examiner from that of the patient. Such separation is crucial as it enhances the understanding of the patient’s behavior in conversational contexts by isolating their verbal responses, which are then analyzed for potential linguistic abnormalities. The audio segments identified through diarization are subsequently transcribed into text using Google’s state-of-the-art transcription technologies (see Fig. 1). This conversion facilitates a detailed examination of the social language used by the patient, aiding in the detection of disorder-specific features within their speech. (2) *Language pattern analysis using GPT*. In this framework, GPT is utilized not only to diagnose ASD but also to identify specific language disorder characteristics that are indicative of ASD. Structured prompts are prepared to gather key information from examiner-patient dialogues. These prompts are crafted as follows: (a) *Examiner-Patient Dialogue* (EPD): The dialogue text, which includes conversational exchanges between the examiner and the patient, serves as the primary input for GPT. This dialogue is carefully processed to maintain the integrity and context of the interaction, ensuring that all relevant linguistic cues are preserved. (b) *Question design:* To guide the analysis, specific questions are formulated based on the dialogue content. These questions aim to direct GPT’s attention to potential signs of language disorders, such as repetitive phrasing, atypical language use, or disrupted conversational flow. c) Knowledge design: This component incorporates domain-specific knowledge from autism diagnostics, which is used to refine GPT’s responses. By integrating expert knowledge, the model is better equipped to recognize and interpret the subtle nuances that characterize ASD-related language disorders. (d) *Prompt Integration*: The complete prompt for GPT includes the dialogue text, the targeted questions, and the expert knowledge cues. This integrated approach helps in precisely pinpointing disorder characteristics that might be overlooked in a less structured analysis. (3) *Functionality of the response parser*: The response parser is a critical element that processes the outputs from GPT. It performs a key function of (Identifying specific characteristics. *Beyond mere diagnosis,* the response parser identifies specific characteristics of the language disorders. It extracts detailed information about the nature and extent of the linguistic anomalies detected, such as the type of stereotypy or idiosyncrasy in the patient’s speech. (4) *Diagnosis based on ADOS-2 Module 4*: The final classification module evaluates the detected features (F1-F10) based on predefined criteria derived from ADOS-2 guidelines. This module integrates domain knowledge to predict A4 scores (A4 = 0 or A4 > 0) by systematically analyzing the presence and severity of SLDs as follows: (a) If critical features, such as F1 (Echoic Repetition) or F9 (Stereotyped Media Quoting), are identified, the dialogue is classified as A4 > 0 (Label 1). These severe features strongly indicate significant social language impairment. (b) Cumulative Features Rule: If more than two other related features (e.g., F2, F3, etc.) are present, the module predicts A4 > 0 (Label 1). This reflects the cumulative impact of multiple moderate symptoms. (c) Non-SLD Classification: If neither severe symptoms nor a sufficient number of related features are detected, the module predicts A4 = 0 (Label 0). This outcome suggests no notable social language disorder in the dialogue. (d) A4 Classification Output: Based on the above rules, the module outputs the A4 classification results: A4 = 0 (Label 0): No significant social language disorders detected. A4 > 0 (Label 1): Significant social language disorders detected.

### Speaker diarization and transcription for examiner-patient interactions

Accurate separation and transcription of examiner-patient dialogues are essential for analyzing linguistic patterns in ASD diagnosis. In this study, we leverage speaker diarization and transcription technologies to automate this process, addressing the limitations of traditional manual annotation. Speaker diarization involves segmenting audio recordings into distinct sections attributed to individual speakers, ensuring precise identification of patient responses while maintaining the conversational context provided by the examiner.

We employed Google’s role-based diarization system, which extends beyond generic speaker labeling (e.g., “Speaker 1” or “Speaker 2”) by explicitly identifying roles as “Examiner” and “Patient.” This functionality is particularly valuable in ASD diagnostics, as it enables the focused analysis of patient-specific language behaviors. Following diarization, the segmented audio files were transcribed into text, creating a structured dataset for linguistic feature extraction and SLD analysis. The workflow includes three key steps: (1) Audio Segmentation: Long recordings were divided into segments based on diagnostic scenarios, such as picture descriptions or abstract reasoning tasks. This segmentation ensures targeted analysis for each task. (2) Speaker Role Labeling: Using Google’s Medical-Conversation Model, the diarization system identified and labeled speaker roles within the conversation, preserving the dynamic interplay between examiner prompts and patient responses. (3) Speech-to-Text Transcription: Each audio segment was transcribed into text with speaker-specific annotations, forming a reliable foundation for subsequent computational analysis.

This automated approach not only reduces the manual workload but also ensures consistent and scalable processing of examiner-patient dialogues, laying the groundwork for robust ASD diagnostic models. By integrating diarization and transcription into a unified pipeline, we streamline the analysis of conversational interactions while maintaining alignment with clinical diagnostic frameworks like ADOS-2.

### Diagnosing language disorders associated with autism via LLM

This subsection details the methodology used to employ LLMs, including GPT and Gemini, for diagnosing SLDs in individuals with ASD. The approach leverages a structured prompt to analyze dialogues between examiners and patients, determining the presence of communicative impairments characteristic of ASD.

The process of prompt design for LLMs involves several parts. In Part 1, Examiner-Patient Dialogue (EPD), the input to LLMs included the transcribed dialogue between the examiner and the patient, presenting the conversational context needed for assessment. In Part 2, Question (Q), following the dialogue, LLMs were asked: “Based on the above conversation between the examiner and the patient, please categorize if any observed SLDs for the patient. Answer only ’Yes’ or ’No’." This question aimed to elicit a definitive response based on the dialogue’s content, focusing solely on the presence or absence of disorder indicators. The prompts used in this study were manually designed based on clinical expertise and ADOS-2 Module 4 guidelines, without any optimization or tuning on the dataset. A fixed prompt template was applied across all subjects and scenarios.

The responses from LLMs were parsed to determine the presence of SLDs. The decision process was as follows: 1) *Response parser*: Each response from GPT, indicating either affirmation ("Yes") or negation ("No"), was analyzed to ascertain whether the patient exhibited symptoms of SLDs based on the dialogue provided. The parser specifically looked for expressions of affirmation or negation concerning the presence of communicative impairments. 2) **Diagnosis Determination:** For each subject, a diagnosis of an SLD was considered positive if there was at least one scenario where GPT affirmed the presence of SLDs ("Yes"). Conversely, if all scenarios resulted in a “No" from GPT, the subject was not considered to have SLDs as per the dialogues analyzed.

By integrating LLMs’ analytical capabilities, this methodology refines the diagnostic process for social language disorders in ASD, enhancing both the efficiency and accuracy of assessments. This approach not only supports clinicians by providing a reliable diagnostic tool but also contributes to the broader field of psycholinguistics by offering insights into the communicative impairments often seen in ASD.

### Identifying language disorder features associated with ASD via GPT

This subsection elaborates on the methodology employed to harness GPT for identifying specific language disorder features associated with ASD, guided by expert knowledge integrated from the ADOS-2, Module 4. The approach utilizes a comprehensive list of language disorders designed around the nuanced communication requirements and symptoms observed in verbally fluent adolescents and adults.

To facilitate the extraction of these features of language deficits using GPT, a specific prompt structure is utilized, as shown in “Prompt 2” (Fig. 1). The prompt was organized into three parts to optimize the analysis, including Part 1: Examiner-Patient Dialogue (EPD), Part 2: Question (Q) - ..., and Part 3: Knowledge (K) - “Overview of the 10 features of social language disorders identified by ADOS-2 examiners, as shown in the column ’Explanation’ in Table 4.

This structured prompt design guides GPT to analyze the transcribed conversations and categorize language deficits, enhancing the PPV of diagnostics based on observed linguistic patterns.

GPT’s responses were analyzed to determine the presence and types of SLD features as follows: (1) Response parser: The parser reviewed GPT’s responses, which involved multiple labels corresponding to the 10 predefined SLD features. Each piece of dialogue could yield several labels, reflecting the multi-dimensional nature of language disorders. (2) Feature classification: Each response was predicted into multiple categories, constituting a multi-label classification task. This approach allowed for a comprehensive assessment of the patient’s language abilities, identifying multiple SLD features from a single excerpt of dialogue.

The multi-label classification approach offers significant advantages in the diagnosis of SLDs. By categorizing dialogue into multiple SLD-related features, GPT enables a comprehensive analysis of the patient’s communicative impairments, providing a nuanced understanding of their specific challenges. This detailed insight is critical for ensuring accurate diagnoses and helps clinicians identify the precise nature of the language deficits. Furthermore, recognizing distinct disorder features allows for the development of targeted intervention strategies, enabling clinicians to design personalized and effective treatments that address the unique difficulties faced by each patient.

Utilizing GPT to identify and classify language disorder features via a structured multi-label classification approach significantly refines the diagnostic capabilities in ASD assessments. This methodology not only enhances the accuracy of the diagnoses but also deepens the understanding of the patient’s specific communicative deficits, facilitating the development of targeted therapeutic strategies.

## Data Availability

The code accompanying this research is publicly available on GitHub: https://github.com/cbhu523/chatgpt_ASD_diagnosis.

## Code availability

The code accompanying this research is publicly available on GitHub: https://github.com/cbhu523/chatgpt_ASD_diagnosis.
